# Dissecting the Role of the Hole-Transport Layer in Cu_2_ AgBiI_6_ Solar Cells: An Integrated Experimental and Theoretical Study

**DOI:** 10.1021/acs.jpcc.4c01871

**Published:** 2024-05-31

**Authors:** Basheer Al-Anesi, G. Krishnamurthy Grandhi, Adriana Pecoraro, Vipinraj Sugathan, Ana Belén Muñoz-García, Michele Pavone, Paola Vivo

**Affiliations:** † Hybrid Solar Cells, Faculty of Engineering and Natural Sciences, Tampere University, P.O. Box 541, FI-33014 Tampere, Finland; ‡ Department of Physics “Ettore Pancini”, University of Naples Federico II, Comp. Univ. Monte Sant’Angelo, 80126 Naples, Italy; § Department of Chemical Sciences, University of Naples Federico II, Comp. Univ. Monte Sant’Angelo, 80126 Naples, Italy

## Abstract

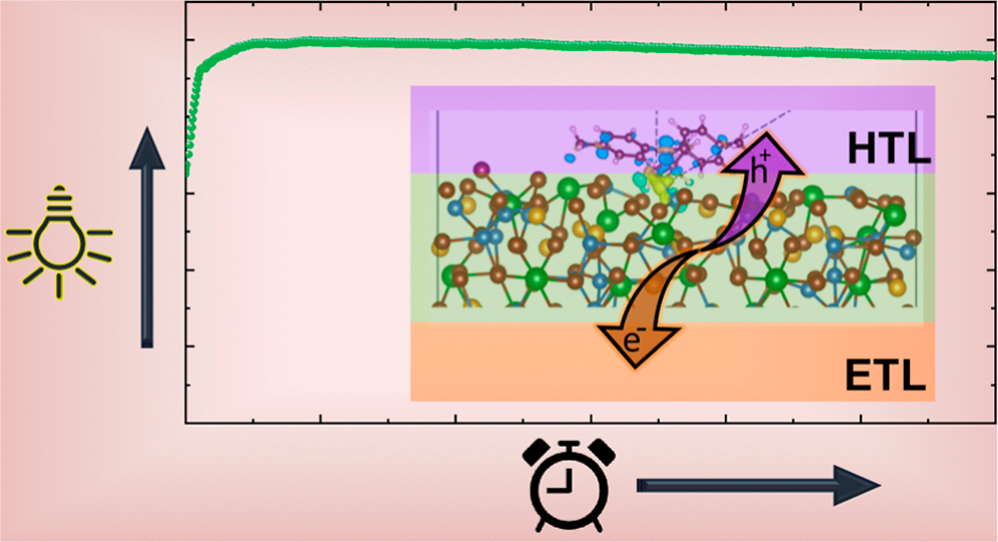

Perovskite-inspired materials (PIMs) provide low-toxicity and air-stable photo-absorbers for several possible optoelectronic devices. In this context, the pnictogen-based halides Cu_2_AgBiI_6_ (CABI) are receiving increasing attention in photovoltaics. Despite extensive studies on power conversion efficiency and shelf-life stability, nearly no attention has been given to the physicochemical properties of the interface between CABI and the hole transport layer (HTL), which can strongly impact overall cell operations. Here, we address this specific interface with three polymeric HTLs: poly(*N*,*N*′-bis(4-butylphenyl)-*N*,*N*′-bis(phenyl)benzidine) (poly-TPD), thiophene-(poly(3-hexylthiophene)) (P3HT), and poly(bis(4-phenyl)(2,4,6-trimethylphenyl)amine) (PTAA). Our findings reveal that devices fabricated with poly-TPD and P3HT outperform the commonly used Spiro-OMeTAD in terms of device operational stability, while PTAA exhibits worse performances. Density functional theory calculations unveil the electronic and chemical interactions at the CABI–HTL interfaces, providing new insights into observed experimental behaviors. Our study highlights the importance of addressing the buried interfaces in PIM-based devices to enhance their overall performance and stability.

## Introduction

Lead halide perovskite (LHP) solar cells are among the most promising emerging photovoltaic (PV) technologies due to their low-cost processing and impressive power conversion efficiency (PCE) values exceeding 26%.^1^ Nevertheless, the significant concerns regarding recycling, arising from the water-soluble and toxic Pb^2+^ in LHPs, are among the major roadblocks to the technological competitiveness of LHP solar cells.^2−5^ Recently, air-stable perovskite-inspired materials (PIMs), particularly the pnictogen-based halide candidates comprising group VA cations Bi(III) and Sb(III), have emerged as low-toxicity alternatives for LHPs, with applications in PVs^6^ and beyond.^7−11^ The reported efforts on PIMs demonstrate the significantly lower performance of PIM-based solar cells (so far limited to 5–6%^12,13^) than the LHP counterparts. However, further advancements toward the theoretical PCE limit approaching 25%^14^ are realistic, given that the research on PIMs is in its early infancy. Efforts to improve the PIM cell efficiencies and their shelf-life stability are often reported, yet the operation stability of these devices has been, so far, almost completely ignored.^10^ On the other hand, the long-term stability under realistic operation conditions^15^ is a key determining factor for the commercial viability of any PV technology. Recent reports on Bi- and Sb-based PIM devices indicate low operational stability, with a *T*_80_ lifetime (i.e., the time taken for their initial PCE to drop to 80%) of just a few hours.^10,16^ It is remarkable that the first examples of solar cells based on LHPs also demonstrated comparable operational instability.^17^ However, thanks to the extensive material and device engineering optimization strategies actively being developed during the years, the current state-of-the-art LHP devices demonstrate 2000hours of stable operation under thermal stress.^18^ This suggests that the limiting factor affecting the stability of PIM-based devices should be carefully examined in order to develop new methods to overcome the performance losses during the operation. The operational stability of PV cells is typically influenced by the chemical composition of the light-harvesting layer, the presence of defects, the selection of the charge transport materials and the interfaces they form with the active layer, the electrodes, and the aging conditions (i.e., illumination, bias, temperature variation in the air, oxygen, and moisture).^15,19^ One extremely relevant parameter to be considered in the case of PIMs is the typical low quality of their films, involving a large number of grain boundaries and high surface density. Indeed, such defects have been identified as the main cause of their performance loss due to their degradation as well as the impairment of other device layers through ion migration under operational conditions.^20−22^ In this context, understanding and engineering the interaction between the exposed upper surface of the active layer and hole transport layer (HTL), i.e., the PIM-HTL buried interface, can be the key to prolonging the device’s lifetime.

Cu_2_AgBiI_6_ (CABI), a Bi-based metal iodide PIM, has recently drawn a great deal of attention due to its impressive long-term environmental stability, direct band gap of ∼2 eV, high absorption cross-section, and easy exciton disassociation, which make CABI suitable for PVs and other optoelectronic applications.^9,10,23−27^ However, only a modest PCE of 0.43% was initially achieved for CABI-based solar cells,^26^ leaving a large room for efficiency improvement. Since then, different optimizations (surface passivation, compositional engineering, processing conditions, and electrodes) have been proposed to boost the PCE of CABI solar cells. In addition, various HTLs have been screened to identify the best candidate materials to achieve high device efficiency. Pai et al. studied the influence of small molecule and polymeric HTLs on the PV parameters of CABI mesoscopic devices and found that Spiro-OMeTAD (2,2′,7,7′-tetrakis(*N*,*N*-di-*p*-methoxyphenylamine)-9,9′-spirobifluorene) leads to the highest device performance.^27^ In another report, Zhang et al. also demonstrated that Spiro-OMeTAD, compared to thiophene-(poly(3-hexylthiophene)) (P3HT), enables higher efficiency in planar CABI solar cells.^23^ Nevertheless, none of these earlier reports has investigated the role of HTL on the device stability aspects, i.e., whether Spiro-OMeTAD is the optimal HTL choice also in terms of device’s lifetime.

In this study, we elucidate the influence of the CAB|HTL buried interface on the performance and stability of CABI solar cells. To this aim, three polymeric HTLs, namely, poly(*N*,*N*′-bis(4-butylphenyl)-*N*,*N*′-bis(phenyl)benzidine (poly-TPD), poly(bis(4-phenyl)(2,4,6-trimethylphenyl)amine (PTAA), and P3HT are chosen. Poly-TPD leads to the highest PCE among the three polymeric HTLs, and P3HT leads to the highest current values, as elucidated by a combined experimental [photoluminescence (PL)) and theoretical (electronic band structure calculations) study. The polymeric HTLs guarantee stable shelf-lifetime trends for more than 1000 h, unlike Spiro-OMeTAD. The comparison of the surface hydrophobicity of these different HTLs explains this stability difference. Finally, the operational stability trends of CABI devices employing polymeric HTLs are monitored. These trends are not the same for all three HTLs, in contrast to their comparable shelf-lifetime trends. To understand this discrepancy, we investigated the binding nature and strength between the surface of CABI and HTL molecules with the help of density functional theory (DFT) calculations.

## Results and Discussion

**Figure 1:**
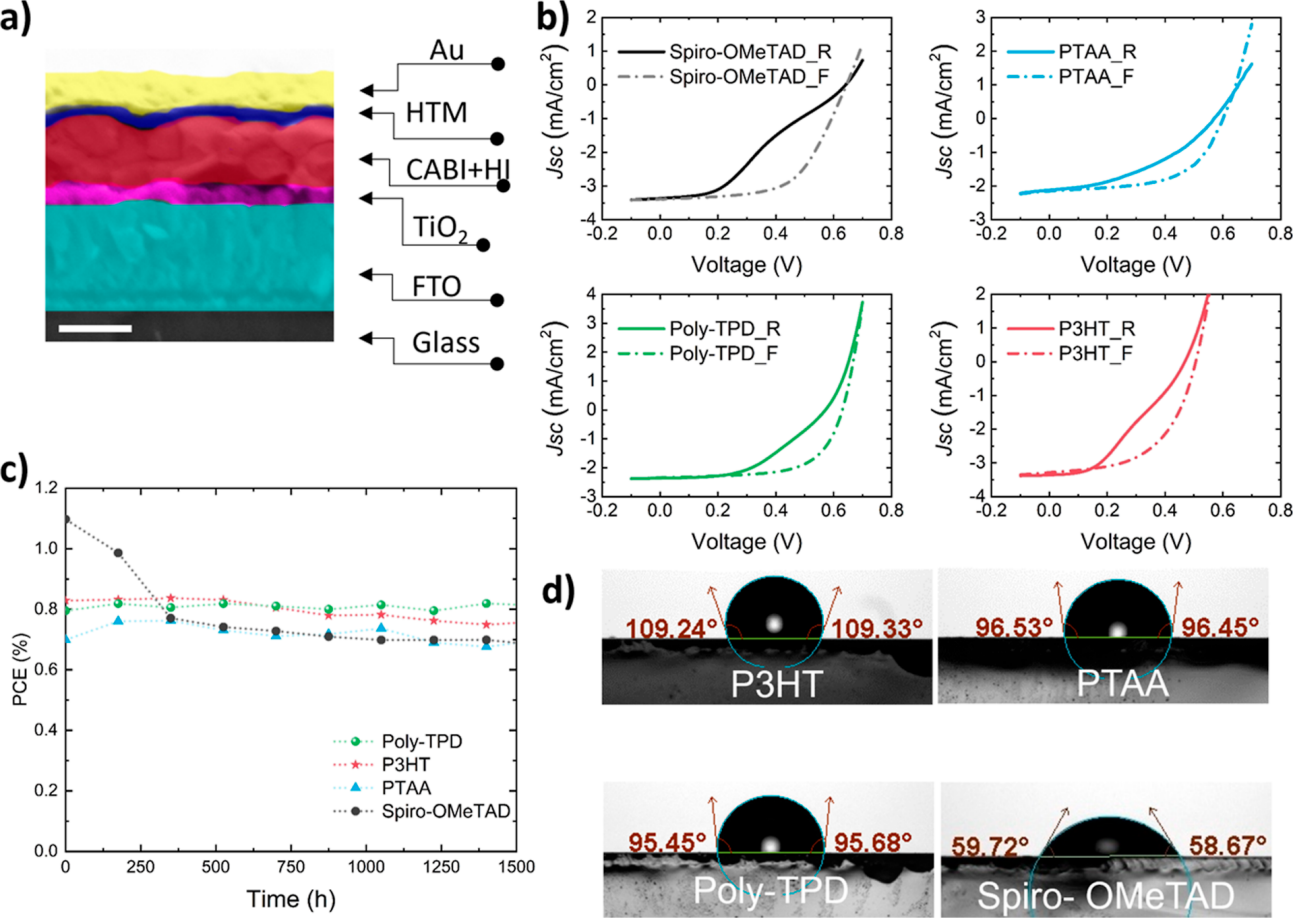
(a) Cross-sectional SEM image of a glass|FTO|c-TiO_2_|CABI|HTL|Au device (scale bar is 300 nm). (b) *J*–*V* curves (forward and reverse bias scans) of the polymer and Spiro-OMeTAD HTL-based champion devices under 1 sun illumination. (c) Shelf-life stability of the unencapsulated polymer HTL- and Spiro-OMeTAD-based solar cells. (d) Water contact angle (WCA) measurement outcomes for polymeric HTLs, and doped Spiro-OMeTAD films atop CABI film.

All the solar cells were fabricated in an n–i–p planar architecture that comprises “FTO|c-TiO_2_|CABI|HTL|Au” (c-TiO_2_ denotes compact TiO_2_) device stacking. Figure 1a illustrates a representative cross-sectional scanning electron microscopy (SEM) image of a polymer HTL-based device. Top-view SEM images of CABI and polymeric HTLs on top of the CABI layer are presented in Figure S1, which demonstrates the successful coverage of the surface of the CABI by the HTLs. The optimized thicknesses of poly-TPD, P3HT, and PTAA are ∼40, ∼80, and ∼10 nm, respectively. CABI, the light-harvesting active layer, was deposited in air by a two-step spin-coating process as reported earlier.^10,26^ The crystallization of the CABI layer in the presence of a small amount of hydroiodic acid (HI) (1.5 vol %) ensured improved surface morphology.^10^

The current density–voltage (*J*–*V*) (forward and reverse bias scans) curves of the champion devices of CABI fabricated using poly-TPD, PTAA, P3HT, and Spiro-OMeTAD HTLs are shown in Figure 1b. Figure S2 shows the statistical distributions of the PV parameters of the three types of devices. External quantum efficiency (EQE) spectra and the corresponding integrated short-circuit density (*J*_SC_) values of the best-performing devices are presented in Figure S3. The *J*_SC_ values of the devices acquired from *J*–*V* measurements closely match those calculated from their EQE spectra with a minor deviation of <3% (Table 1).

**Table 1:** Photovoltaic Parameters of the Polymeric HTL-Based Devices and Current Density Extracted from EQE

HTM	PCE (%)	FF (%)	*J*_sc_^IV^ (mA/cm^2^)	*V*_oc_ (V)	*J*_sc_^EQE^^a^ (mA/cm^2^)
poly-TPD	0.83 ± 0.06 (0.94)^b^	65 ± 0.8 (64)	2.31 ± 0.1 (2.35)	0.62 ± 0.01 (0.63)	2.29
P3HT	0.83 ± 0.04 (0.90)	54 ± 0.7 (55)	3.00 ± 0.2 (3.29)	0.50 ± 0.01 (0.50)	3.31
PTAA	0.68 ± 0.03 (0.74)	55 ± 1.8 (57)	2.06 ± 0.1 (2.15)	0.58 ± 0.01 (0.60)	2.11

a *J*_sc_^EQE^ is extracted from EQE spectra.

b The values in brackets represent the parameters of the champion devices.

While poly-TPD HTL led to a higher PCE of 0.94% for CABI-based solar cells among the three polymeric HTLs, the Spiro-OMeTAD-based devices had delivered the highest PCE of ∼1.3% for CABI devices in a planar architecture.^10^ Nevertheless, Spiro-OMeTAD-containing CABI devices are known to exhibit a larger hysteresis between the reverse and forward voltages, Nevertheless, Spiro-OMeTAD-containing CABI devices are known to exhibit a larger hysteresis between the reverse and forward voltage *J−V* scans. On the other hand, the polymeric HTLs ensured a lower *J*–*V* hysteresis for the CABI devices (see Figure 1b). More remarkably, the polymeric HTL-based CABI solar cells demonstrated enhanced air-stability.

We monitored the shelf-life stability of unencapsulated polymeric HTL-based devices up to 1440 h in the air with a relative humidity (RH) of 50% at 25 °C. The stability trends of the polymeric HTL-based devices in comparison with that of Spiro-OMeTAD containing cells are presented in Figure 1c. While the poly-TPD-based device exhibits a superior stability with nearly no loss of the initial PCE after ∼1400 h, PTAA and P3HT HTLs led to a reduced PCE of CABI devices by ∼4 and 10%, respectively, during the same period. It should be noted that the most stable devices in air are those based on triphenylamine HTLs (i.e., poly-TPD and PTAA). On the contrary, the control Spiro-OMeTAD-based device gradually degraded and experienced a ∼40% PCE loss after 1440 h, predominantly due to a reduction in the *J*_SC_. Although the initial PCE of 1.1% of the Spiro-OMeTAD containing solar cell was higher than those of the polymeric HTLs (∼0.7 to 0.83%), after 1500 h of storage in the air, the nearly unchanged PCE trends of poly-TPD and P3HT-based devices ensured that their final PCE values (∼0.75 to 0.82%) are higher compared to that of the degraded Spiro-OMeTAD-based device (0.7% PCE). The *J*_SC_ loss in the Spiro-OMeTAD containing device can be attributed to a gradual and severe degradation of both the light-harvesting layer (i.e., CABI) and Spiro-OMeTAD HTL due to the adsorption of the moisture from the environment by the LiTFSI hygroscopic dopant present in the HTL. To verify this, we measured the water contact angles (WCAs) of glass|CABI|HTL films. As expected, the three dopant-free polymer HTL films showed WCAs in the >96.0 to –109.0° range (Figure 1d), which are significantly higher values than those obtained in the case of doped Spiro-OMeTAD (<59.0°). The hydrophobic nature of the polymeric HTLs is beneficial to protect CABI solar cells under humid ambient conditions. According to the above results, unlike Spiro-OMeTAD, the polymeric HTLs guaranteed a high shelf-lifetime for the CABI solar cells. Thus, the rest of the article is dedicated to getting insights into the performance and operational stability of the three polymeric HTL-based devices.

**Figure 2:**
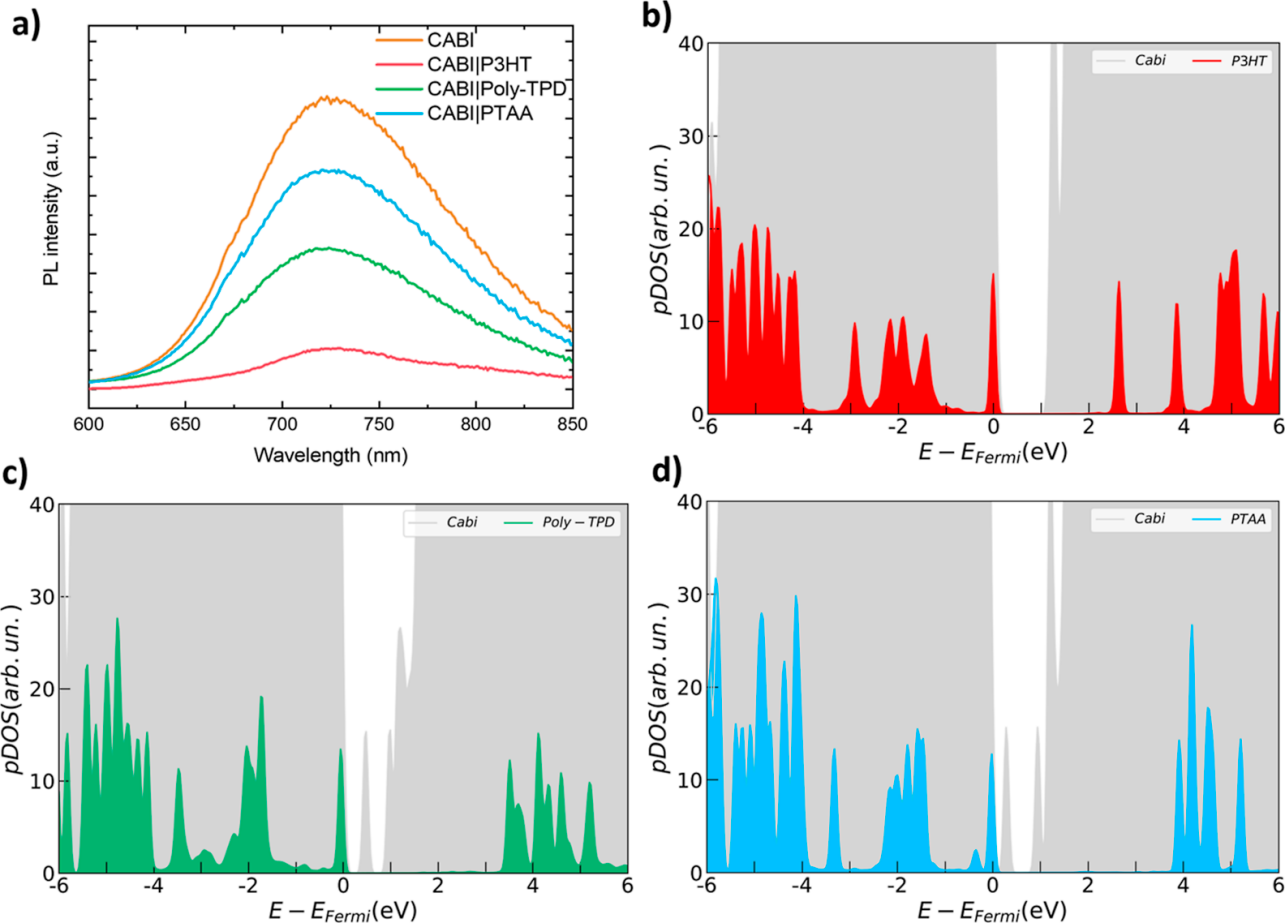
(a) PL spectra (λ_ex_ = 405 nm) of CABI, CABI|P3HT, CABI|Poly-TPD, and CABI|PTAA films on a glass substrate. The density of states projected into CABI and HTL contributions for the three investigated interfaces (pDOS), (b) CABI|P3HT, (c) CABI|Poly-TPD, and (d) CABI|PTAA. All pDOS have been calculated at HSE06 level of theory on PBE-TS-optimized interface structures.

The higher performance of poly-TPD based devices may be attributed to the high hole mobility (4 × 10^–4^ cm^2^ V^–1^ s^–1^)^28^ of poly-TPD as well as to the well-aligned highest occupied molecular orbital (HOMO) of poly-TPD (−5.2 eV)^29^ with the valence band (VB) of CABI (−5.25 eV),^27^ in turn enabling high fill factor (FF) and open-circuit voltage (*V*_OC_). The other triphenylamine-based HTL, PTAA resulted in *V*_OC_ values comparable to the case of poly-TPD. However, the low *J*_SC_ values of the PTAA-based devices (the hole mobility of PTAA is one order of magnitude lower than that of poly-TPD, on the order of 10^–5^ cm^2^ V^–1^ s^–1^)^30^ resulted in their lowest average PCE of 0.68%. On the other hand, the P3HT-based devices yielded low *V*_OC_ values, which may be attributed to undesired energy-level misalignment between P3HT and CABI (HOMO of P3HT and VB of CABI lie at −4.8 and −5.25 eV, respectively).^27^ However, the P3HT-based devices still delivered a maximum PCE of 0.9% due to their high *J*_SC_ values, consistently with previous studies.^23,27^ Consequently, P3HT HTL enabled significantly higher EQE values in the 300–700 nm wavelength range for CABI devices than poly-TPD and PTAA HTL devices. The steady-state PL quenching measurements were carried out on CABI|HTL films to investigate the hole extraction ability of the polymeric HTLs (Figure 2a). The broad emission spectrum of CABI peaked at 723 nm, which closely matches that of CABI (i.e., 720 nm) from our previous studies.^31^ P3HT coating led to higher quenching of the emission intensity of poly-TPD and PTAA atop CABI. This suggests better hole charge extraction in the P3HT case, i.e., a rapid hole injection from the CABI layer to the P3HT, which clearly reflects the enhanced current density of P3HT HTL-based CABI devices.

Electronic interaction at the CABI|HTL interface has been analyzed via HSE-TS density of states (DOS) resolved into CABI and HTL contributions (pDOS), as reported in Figure 2b–d. pDOS provides a qualitative indication of the energy alignment between HOMO of HTL and VB maximum (VBM) of CABI, fundamental to assessing the thermodynamic driving force for photogenerated hole extraction. For efficient hole extraction, the HOMO of the HTL should lie at a higher energy than the VBM of the active material. However, the band offset between HOMO and VBM should be low so as to minimize the *V*_OC_ losses, as observed in the case of poly-TPD|CABI vs P3HT|CABI. Both poly-TPD and PTAA show additional localized mid-gap states belonging to CABI atoms (Figure 2b–d). The presence of such states can trigger undesired recombination phenomena, suggesting a more efficient charge transfer for the P3HT molecular system, as also suggested by both PL quenching data (Figure 2a) and high *J*_SC_ values of the corresponding devices (Table 1).

**Figure 3:**
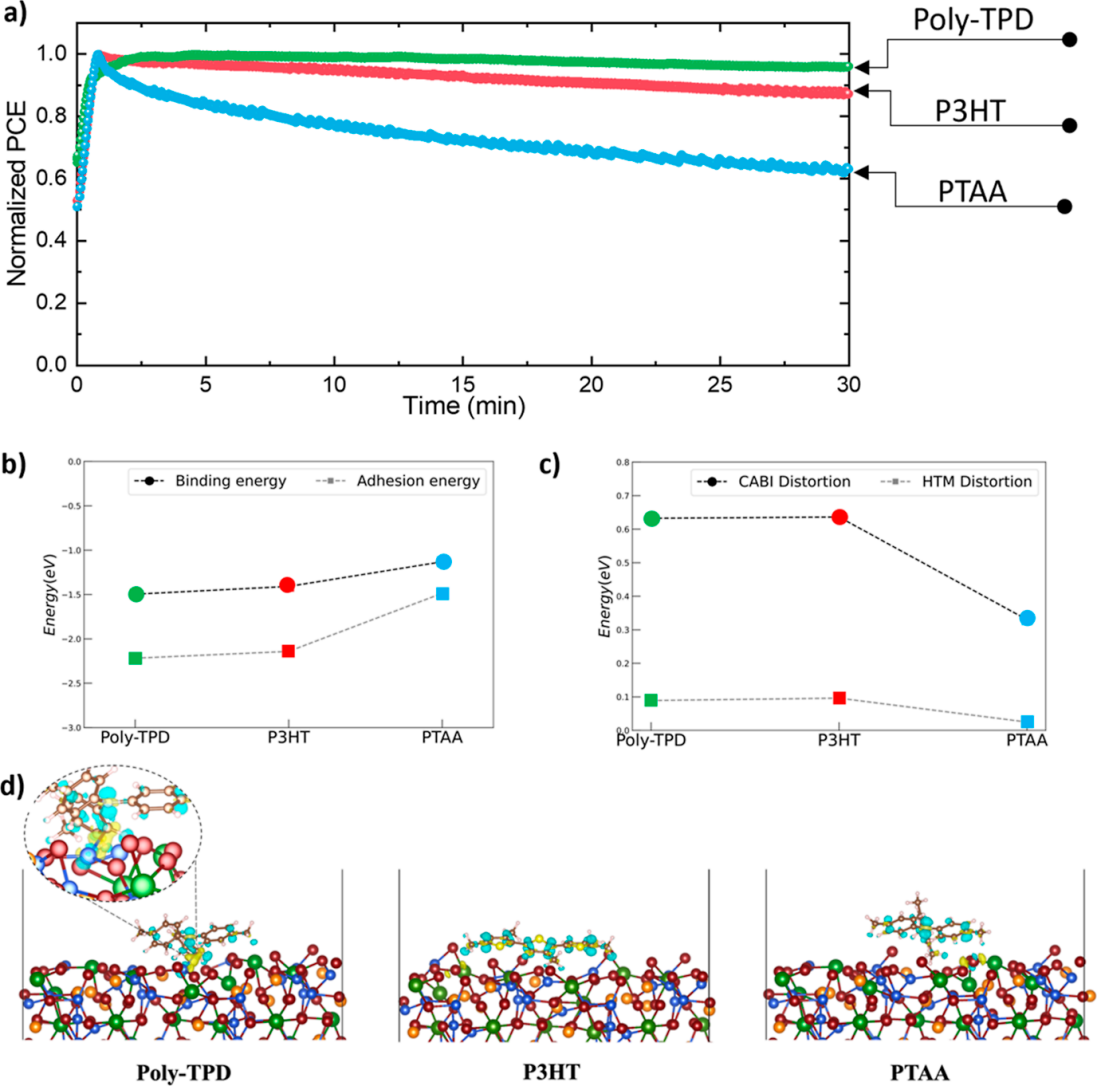
(a) Operational stability test of polymeric HTL-based CABI photovoltaic devices. The devices were aged in the air (50% RH) at 25 °C under continuous AM 1.5 G illumination at MPP. (b) Binding and adhesion energies and (c) structural distortion contributions calculated at HSE06+TS level of theory for each interface model. (d) Lateral views of the relaxed CABI|HTL interfaces (left poly-TPD, central P3HT, and right PTAA). Yellow and blue regions denote charge accumulation and depletion zones represented using a 0.005 a.u. iso-surface value. Color legend for atomic spheres: Cu-blue; Bi-green; Ag-orange; I-purple; C-brown; H-light pink; S-yellow; and N-light blue.

To understand how the different polymeric HTLs influence the operational stability of the corresponding CABI solar cells, the unencapsulated devices were aged for 30 min in air under continuous 1 sun illumination (AM 1.5 G; 100 mW cm^–2^) at the maximum power point (MPP). The normalized PCE trends of the devices as a function of the illumination time are shown in Figure 3a. The poly-TPD-based device showed excellent operational stability by preserving 97% of its initial efficiency. P3HT HTL led to a drop in ∼14% of the initial PCE of the CABI device. The PTAA-based device exhibited a marked drop (37%) in the initial PCE, with a sharp fall in PCE even during the first few minutes of MPP tracking. It is worth mentioning that the operational stability of the PTAA-based device is significantly lower than that for the devices employing the other two HTLs, in contrast to the comparable shelf-life stability trends of all the three HTLs. The corresponding MPPT trends with the absolute PCE values are presented in Figure S4. This observation, in turn, suggests that the nature of interactions at the CABI|HTL interface plays a role in the operational stability of CABI solar cells, and the shelf-lifetime will not necessarily be mirrored into the operational stability of PV devices.^16^ It should be noted that many additional parameters, such as the instability of each constituent layer and the interfaces between layers of the devices under different external stressors (i.e., illumination, bias, temperature variation in air, oxygen, and moisture), and a very high number of defects in the active layer, may also contribute to the operational instability of CABI and other PIM-based devices. Such multifaceted study is beyond the scope of this present work. However, the current study on elucidating the role of HTL is a step forward toward understanding and mitigating the operational instability of CABI (and other PIMs)-based PV devices. In addition, the MPPT measurements should be conducted in controlled environmental conditions, such as an inert atmosphere and a fixed temperature, following ISOS protocols defined for perovskite solar cells to evaluate the actual device decay parameters, such as *T*_80_ and lifetime.

To probe into the CABI|HTL interface for the three types of devices, we performed a DFT-based investigation of the interfaces between the (110) surface of CABI and the different HTLs, P3HT, PTAA, and poly-TPD (see Structural Models in the Supporting Information for details). We assessed the structural stability of the three interface models in terms of the binding energies (*E*_b_) calculated as
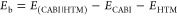
1where the three terms represent the total energy of the optimized interface structure (*E*_(CABI|HTM)_) and the energies of the isolated CABI substrate (*E*_CABI_) and of the HTL (*E*_HTM_), respectively. A further decomposition of the binding energy, in terms of the pure electrostatic adhesion energy (*E*_a_) and structural distortion contributions, has been performed for all the models according to eq 2
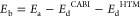
2where the adhesion energy (*E*_a_) is the total energy difference between the fully relaxed interface and its constituents (HTL and surface) at the interface geometry while *E*_d_^CABI^ and *E*_d_^HTM^ are the differences between the energies of each system at the interface geometry and those at their fully relaxed configuration. Figure 3 shows the behaviors of these quantities for different HTLs. All the energy contributions have been evaluated at the HSE06-TS level of theory on top of PBE-TS fully relaxed interfaces. Very close binding energy values are obtained for P3HT and poly-TPD, only differing by 0.08 eV, with poly-TPD forming the most favorable interface (Figure 3b). Poly-TPD interface shows strong binding to the surface of CABI, and the strong interaction is confirmed by the massive charge reorganization mostly localized along the Cu–C bond where a charge accumulation region is observed that suggests the formation of a covalent bond between the two atoms (Figure 3d). We hypothesize that strong binding of the poly-TPD on the surface of CABI minimizes the ion movement and surface defect formation under operational conditions, hence prolonging the device’s stability. In the case of P3HT, the interaction appears to primarily result from electrostatic interaction, which is facilitated by the flat shape of the molecule, which maximizes the contact area with CABI. On the other hand, the highest binding energy has been found for the PTAA|CABI interface, which is about 0.3 eV higher than the other two polymers. PTAA-based interfaces show similar electrostatic interactions to P3HT; however, the smaller CABI|PTAA surface contact explains the less favorable binding energy value, which, in turn, explains the poor operational stability of the PTAA-employed CABI solar cells. The binding energy trend (poly-TPD|CABI > P3HT|CABI ≫ PTAA|CABI) between the three interfaces matches that of the operational stability of the devices fabricated using the three HTLs (Figure 3a). Furthermore, the comparison between the adhesion and distortion contributions reveals a predominant electrostatic interaction among the HTL molecules and CABI’s surface (Figure 3c). An HTL|CABI interaction strength can be visualized by charge density difference sketches, as displayed in Figure 3d. The tight and favorable binding of the poly-TPD and P3HT molecules onto the CABI surface responsible for the improved operational stability of the corresponding solar cells, unlike in the case of CABI|PTAA, is further evident from these charge density models.

## Conclusions

In summary, our investigation addressed the impact of three distinct polymeric HTLs on the overall performance of CABI solar cells. Notably, devices utilizing poly-TPD and P3HT HTLs demonstrated an exceptional operational efficiency. According to first-principles calculations, the enhanced operational stability is attributed to the robust binding of HTL molecules to the CABI surface. Conversely, the inferior performance of PTAA-based devices stems from weak electrostatic interactions at the CABI interface, leading to rapid performance degradation. Our findings underscore the critical role of HTL-CABI binding strength in dictating device degradation pathways and, consequently, long-term performance. We highlight that while device shelf life is a crucial consideration, it does not necessarily guarantee sustained performance under continuous operation. This study emphasizes the significance of investigating and optimizing buried interfaces to enhance long-term performance under real operational conditions. By addressing operational stability alongside power conversion efficiency, we pave the way for future research aimed at mitigating stability issues in CABI and related perovskite-inspired absorber devices. This is essential for ensuring the enduring performance of PIMs and promoting these low-toxicity and inherently stable absorbers for optoelectronic device applications in PVs and beyond.

## Experimental Methods

### Materials and Device Fabrication

Bismuth iodide (BiI_3_), copper iodide (CuI), titanium diisopropoxide bis(acetylacetonate) (TDBA) 75 wt % in isopropanol, dimethylsulfoxide, hydroiodic acid (HI, 57%), chlorobenzene (CB, extra dry, 99.8%), acetonitrile (99.9%), and 4-*tert*-butylpyridine (4-tBP), bis(trifluoromethane)sulfonimide lithium salt (Li-TFSI, 99.95%) were purchased from Sigma-Aldrich. Tris[2-(1*H*-pyrazol-1-yl)-4-*tert*-butylpyridine]cobalt(III)tri[bis(trifluoromethane) sulfonimide] (FK209 Co(III), >98%) was purchased from Dyenamo. Dimethylformamide, silver iodide (AgI), and toluene were purchased from Alfa Aesar. The hole transport materials 2,2′,7,7′-tetrakis(*N*,*N*-di-*p*-methoxy phenylamino)-9,9 spirobifluorene (Spiro-OMeTAD), poly[*N*,*N*′-bis(4-butylphenyl)-*N*,*N*′-bis(phenyl)-benzidine] (poly-TPD), and poly[bis(4-phenyl)(2,4,6-trimethylphenyl)amine] (PTAA) were purchased from Lumtec. Poly(3-hexylthiophene-2,5-diyl (P3HT) was purchased from Rieke Metals. Fluorine-doped tin(IV) oxide (FTO)-coated glass substrates of 2 cm × 2 cm were purchased from Yingkou OpvTech New Energy Technology Co. All the chemicals were used as received.

Pre-etched FTO substrates were sonicated for 15 min in each step with aqueous Mucasol solution (2% v/v), deionized water, acetone, and 2-propanol. The cleaned FTO substrates were dried using a nitrogen gas flow. On the as-prepared FTO substrate, a compact titanium dioxide layer (c-TiO_2_) (thickness ∼ 70 nm) was deposited at 450 °C by spraying TDBA in 2-propanol solution (0.38 M), and then the substrates were sintered (at 450 °C for 1 h) in air.^32^ The CABI precursor was prepared and deposited on top of c-TiO_2_ as shown in previous work.^10^ HTLs used in this work included doped Spiro-OMeTAD (28 mM in CB), P3HT(20 mg/mL in CB), poly-TPD (10 mg/mL in CB), and PTAA (0.5 mg/mL in toluene). The Spiro-OMeTAD was doped with 14.39 μL of TBP, 8.75 μL of LiTFSI (500 mg/mL in ACN), and 14.50 μL of FK209 (300 mg/mL in ACN). This solution was deposited dynamically by spin-coating 80 μL at 1800 rpm for 30 s. P3HT was deposited dynamically by spin-coating 80 μL at 2000 rpm for 30 s. Poly-TPD and PTAA were deposited by spin-coating 80 and 100 μL at 4000 and 5000 rpm for 30 s, respectively. In the case of PTAA, substrates were annealed at 100 °C for 10 min after depositing the PTTA layer. P3HT, poly-TPD, and PTAA-based devices were stored overnight inside the nitrogen-filled glovebox, while Spiro-OMeTAD-based devices were stored overnight in a dry air atmosphere for the oxidation process. Lastly on the next day, on top of the HTM layer, a 100 nm thick gold contact was thermally evaporated using an OPTIvap evaporation system (CreaPhys GmbH) under vacuum (pressure < 10^–6^ mbar) via a metal shadow mask to obtain an active area of each solar cell of 20 mm^2^.

### Characterization

A Keithley 4250 source-monitor unit was used to measure *J*–*V* curves of unencapsulated devices under ambient conditions (RH ∼ 40%, RT = 23 ± 1 °C) under simulated solar radiation illuminated by an AAA-solar simulator (SINUS-70 LED solar simulator from Wavelabs). Calibration of the device to 1 sun intensity (100 mW/cm^2^ irradiance) was performed by using KG5 silicon reference cells. Quantx-300 system (Newport Instruments) was used for the EQE measurements in ambient air with monochromatic light at zero bias light. A silicon solar cell was used to calibrate the system before the measurement. The operational stability test of unencapsulated devices was carried out using a Keithley 4250 source-monitor unit controlled by the MATLAB script under continuous 1 sun illumination at the MPP. The PL spectra of the film samples, excited at 405 nm, were measured within the wavelength range 600–920 nm with a step size of 1 nm by using an FLS1000 PL spectrometer (Edinburgh Instruments Ltd., UK). WCAs of the glass|CABI + HI|HTM films were measured using an Attention Theta Lite optical goniometer (Biolin Scientific AB, Sweden). Top-view SEM images of the films andcross-section SEM image of a glass|FTO|c-TiO_2_|CABI|HTL|Au device was collected using a field emission scanning electron microscope (FE-SEM, Zeiss ULTRA plus, Carl Zeiss, Germany) operated at an acceleration voltage 3 kV.

### Computational Details

All electron spin-polarized DFT^33^ calculations with periodic boundary conditions have been performed using basis set of numerical atom-centered orbitals (NAO)^34^ as implemented in the Fritz Haber Institute Ab Initio Molecular Simulations (FHI-aims) code.^35^ Electrons are described with the light-tier1 basis of NAO and the zero-order regular approximation. The Perdew–Burke–Ernzerhof (PBE)^36^ density functional has been employed for all geometry optimizations, including the Tkatchenko–Scheffler (TS) correction^37^ for dispersion. We employed a 1 × 10^–6^ eV threshold for self-consistency convergence of total energy and a 0.02 eV Å^–1^ threshold for maximum forces for structural relaxation. All calculations have been performed at the Γ point of the Brillouin zone. Mixed occupancy of Bi/Ag/vacancy was simulated via the special quasi-random structure (SQS) approach.^38,39^ Electronic calculations have been refined with single-point calculations with the HSE06 hybrid functional^40^ for projected density of states (pDOS) and formation energies.

## Supporting Information Available

Top-view SEM images, statistical distributions of the PV parameters, EQE spectra, absolute PCE under MPPT condition, and monomeric models of the polymeric HTLs (PDF)
