# Supplementary material for: Dissecting the Role of the Hole-Transport Layer in Cu2 AgBiI6 Solar Cells: An Integrated Experimental and Theoretical Study
Source: J Phys Chem C Nanomater Interfaces. 2024 May 31;128(23):9446–53. doi: 10.1021/acs.jpcc.4c01871 (PMC11182344; doi:10.1021/acs.jpcc.4c01871)
Supplement: Supplementary file 1 — jp4c01871_si_001.pdf [file jp4c01871_si_001.pdf]

Supporting Information (SI)

Dissecting the role of hole-transport layer in Cu_2AgBiI_6 solar cells: an integrated experimental and theoretical study

Basheer Al-Anesi,[a] G. Krishnamurthy Grandhi,[a] Adriana Pecoraro,[b] Vipinraj Sugathan,[a] Ana Belén Muñoz-García,[b] Michele Pavone,[c] and Paola Vivo*,[a]

(a) *Hybrid Solar Cells, Faculty of Engineering and Natural Sciences, P.O. Box 541, FI-33014 Tampere University, Finland.*
(b) *Department of Physics "Ettore Pancini" University of Naples Federico II, Comp. Univ. Monte Sant'Angelo 80126 Naples, Italy.*
(c) *Department of Chemical Sciences, University of Naples Federico II, Comp. Univ. Monte Sant'Angelo 80126, Naples, Italy.*

*Corresponding author, Paola Vivo, paola.vivo@tuni.fi

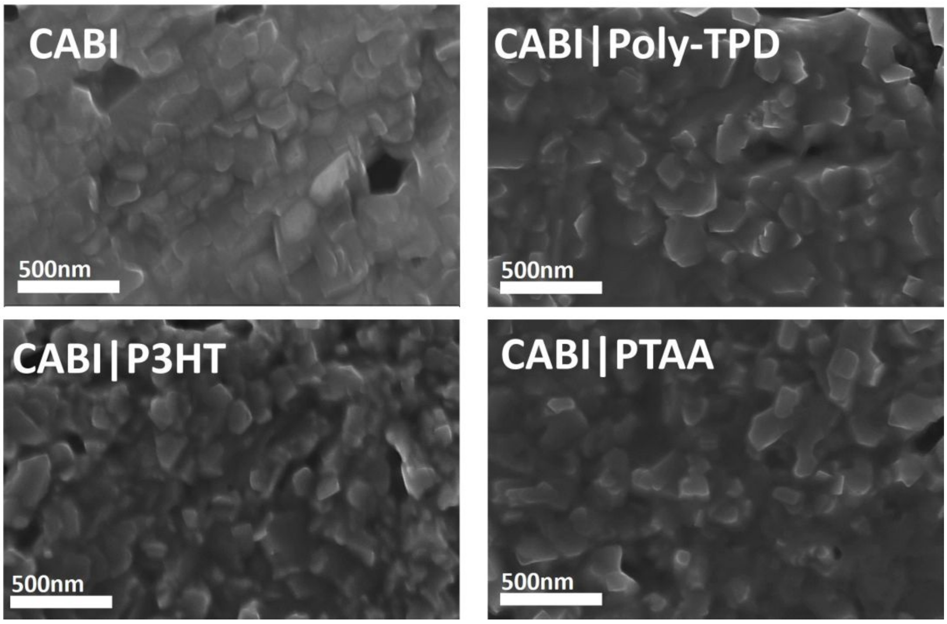

Figure S1. Top-view SEM images of CABI and and polymer HTLs on top of the CABI layer.

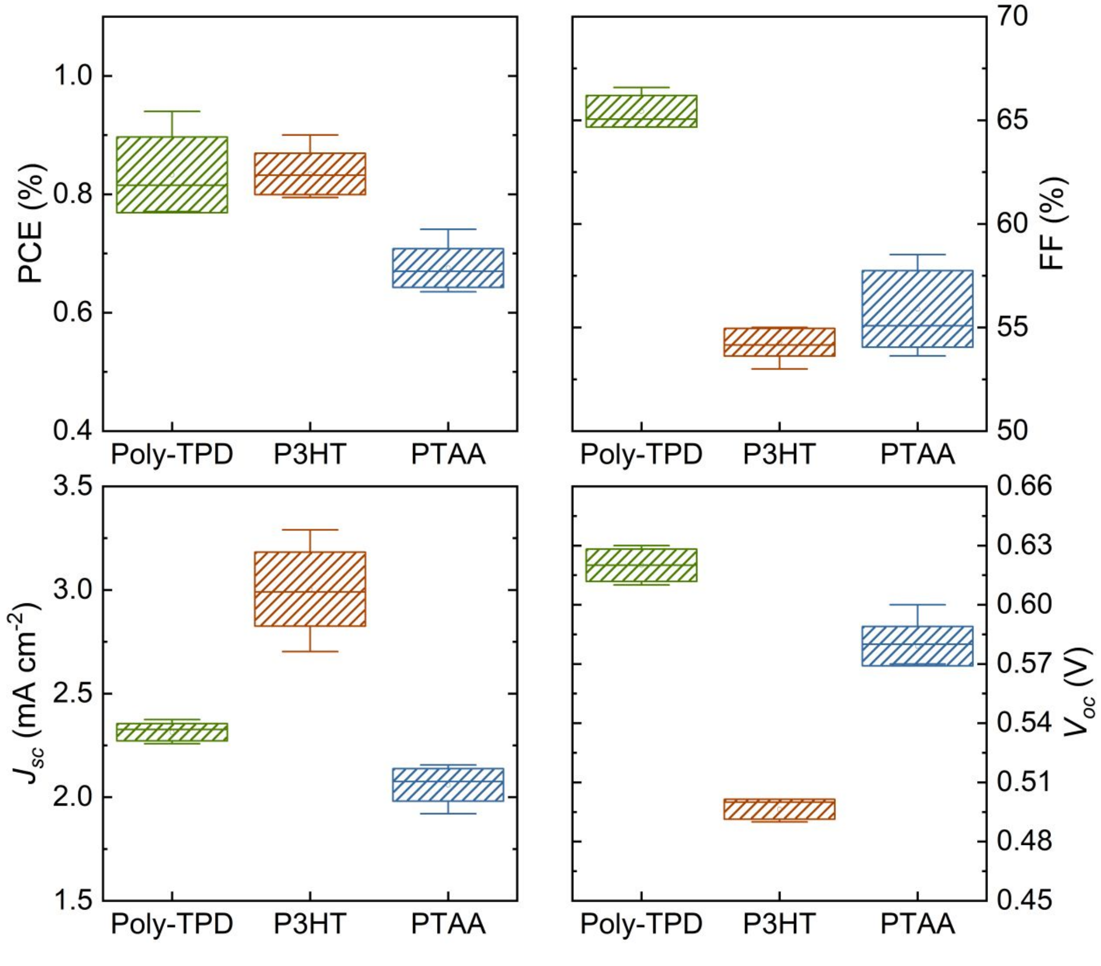

Figure S2. Statistical distributions of the photovoltaic parameters of the polymeric HTLs-based devices.

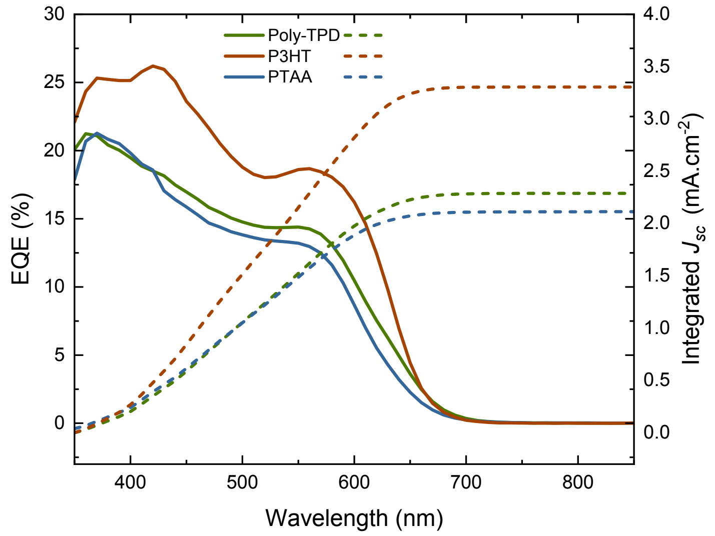

Figure S3. External quantum efficiency (EQE) spectra and the corresponding integrated JSC values of the best-performing polymeric HTLs-based devices.

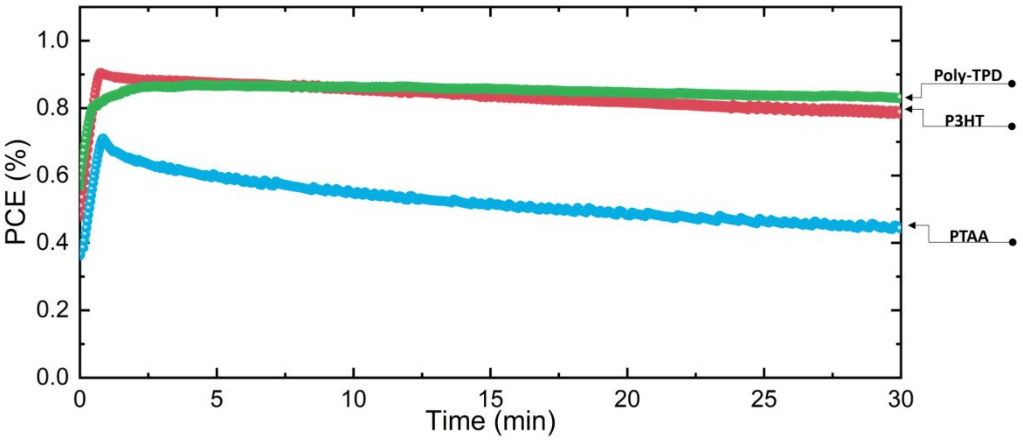

Figure S4. Absolute PCE values of polymeric HTLs-based devices under MPPT condition.

Structural models

The starting point for the computational model is the CABI structure ($R\bar{3}m$ No. 166) provided by Sansom et al.[1] Random distribution and partial occupation of Cu, Bi, Ag atoms and vacant sites was realized through a special quasi-random structure toolkit. The optimized bulk structure was cleaved along the stable (110) surface to realize the slab model.[2] A 2x1x1 supercell was considered to accommodate each HTM and a 20 Å vacuum was considered so to avoid interaction between periodic images. For the three HTMs, namely Poly-TPD, PTAA

and P3HT, a monomeric unit model has been considered. Computational models for HTMs are shown in **Figure S4.**

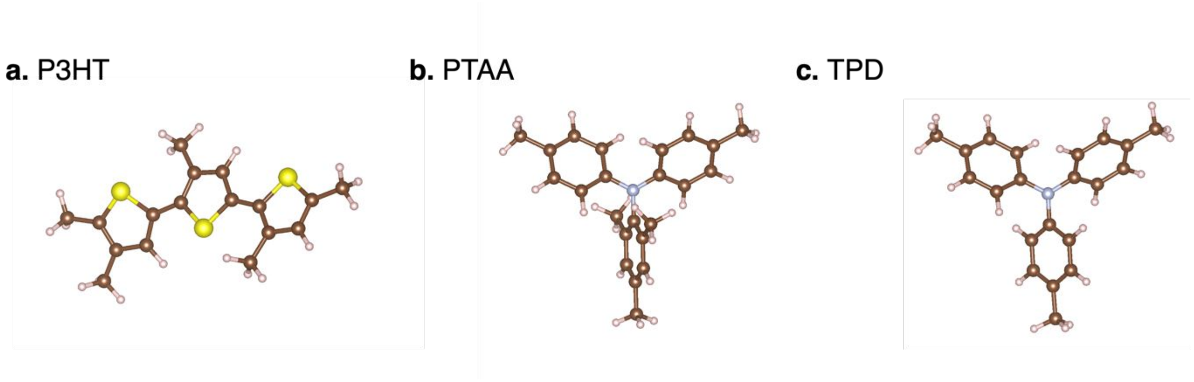

Figure S5. Monomeric models for P3HT, PTAA and TPD, HTMs. Color Legend for atomic spheres: C-brown; H-light pink; S-yellow; N-light blue.

References

1. Sansom, H. C.; Longo, G.; Wright, A. D.; Buizza, L. R. V.; Mahesh, S.; Wenger, B.; Zanella, M.; Abdi-Jalebi, M.; Pitcher, M. J.; Dyer, M. S.; Manning, T. D.; Friend, R. H.; Herz, L. M.; Snaith, H. J.; Claridge, J. B.; Rosseinsky, M. J. Highly Absorbing Lead-Free Semiconductor Cu2AgBiI6 for Photovoltaic Applications from the Quaternary CuI-AgI-BiI3Phase Space. *J. Am. Chem. Soc.* **2021**, *143*, 3983–3992.

2. Grandhi, G. K.; Al-Anesi, B.; Pasanen, H.; Ali-Löytty, H.; Lahtonen, K.; Granroth, S.; Christian, N.; Matuhina, A.; Liu, M.; Berdin, A.; Pecunia, V.; Vivo, P.; Grandhi, G. K.; Al-Anesi, B.; Pasanen, H.; Christian, N.; Matuhina, A.; Liu, M.; Vivo, P.; Ali-Löytty, H.; Lahtonen, K.; Granroth, S.; Berdin, A. Enhancing the Microstructure of Perovskite-Inspired Cu-Ag-Bi-I Absorber for Efficient Indoor Photovoltaics. *Small* **2022**, *18*, 2203768.
